# Continental‐scale dynamics of avian influenza in U.S. waterfowl are driven by demography, migration, and temperature

**DOI:** 10.1002/eap.2245

**Published:** 2020-11-22

**Authors:** Erin E. Gorsich, Colleen T. Webb, Andrew A. Merton, Jennifer A. Hoeting, Ryan S. Miller, Matthew L. Farnsworth, Seth R. Swafford, Thomas J. DeLiberto, Kerri Pedersen, Alan B. Franklin, Robert G. McLean, Kenneth R. Wilson, Paul F. Doherty

**Affiliations:** ^1^ School of Life Sciences University of Warwick Coventry CV4 7AL United Kingdom; ^2^ The Zeeman Institute: Systems Biology and Infectious Disease Epidemiology Research (SBIDER) University of Warwick Coventry CV4 7AL United Kingdom; ^3^ Department of Biology Colorado State University Fort Collins Colorado 80521 USA; ^4^ Graduate Degree Program in Ecology Colorado State University Fort Collins Colorado 80521 USA; ^5^ Department of Statistics Colorado State University Fort Collins Colorado 80521 USA; ^6^ Centers for Epidemiology and Animal Health USDA APHIS Veterinary Services Fort Collins Colorado 80526 USA; ^7^ National Wildlife Disease Program USDA APHIS Wildlife Services Fort Collins Colorado 80521 USA; ^8^ National Wildlife Refuge System US Fish and Wildlife Service Yazoo City Mississippi 39194 USA; ^9^ USDA APHIS Wildlife Services Raleigh North Carolina 27606 USA; ^10^ National Wildlife Research Center USDA APHIS Wildlife Services Fort Collins Colorado 80521 USA; ^11^ Department of Fish, Wildlife, and Conservation Biology Colorado State University Fort Collins Colorado 80521 USA

**Keywords:** avian influenza, bird migration, contact structure, influenza A virus, multi‐scale analysis, network

## Abstract

Emerging diseases of wildlife origin are increasingly spilling over into humans and domestic animals. Surveillance and risk assessments for transmission between these populations are informed by a mechanistic understanding of the pathogens in wildlife reservoirs. For avian influenza viruses (AIV), much observational and experimental work in wildlife has been conducted at local scales, yet fully understanding their spread and distribution requires assessing the mechanisms acting at both local, (e.g., intrinsic epidemic dynamics), and continental scales, (e.g., long‐distance migration). Here, we combined a large, continental‐scale data set on low pathogenic, Type A AIV in the United States with a novel network‐based application of bird banding/recovery data to investigate the migration‐based drivers of AIV and their relative importance compared to well‐characterized local drivers (e.g., demography, environmental persistence). We compared among regression models reflecting hypothesized ecological processes and evaluated their ability to predict AIV in space and time using within and out‐of‐sample validation. We found that predictors of AIV were associated with multiple mechanisms at local and continental scales. Hypotheses characterizing local epidemic dynamics were strongly supported, with age, the age‐specific aggregation of migratory birds in an area and temperature being the best predictors of infection. Hypotheses defining larger, network‐based features of the migration processes, such as clustering or between‐cluster mixing explained less variation but were also supported. Therefore, our results support a role for local processes in driving the continental distribution of AIV.

## Introduction

Surveillance and risk assessments for emerging diseases of wildlife origin are informed by a mechanistic understanding of their spread and distribution in wildlife reservoirs (Cunningham et al. 2017). A fundamental challenge in characterizing these mechanisms is quantifying the relative importance of multiple processes acting across spatial scales (Plowright et al. 2008, Tompkins et al. 2011). Processes acting at local scales may predominate and scale up to influence disease at larger scales. For example, the seasonal aggregation of school children or urban workers is thought to be a dominant driver of directly transmitted, immunizing infections, such as measles (Metcalf et al. 2009, Bharti et al. 2011). Alternatively, larger‐scale processes such as climate variability or seasonal movements can also drive disease dynamics (Wesolowski et al. 2012, Metcalf et al. 2017). Understanding how these processes interact and scale is essential to designing pathogen‐specific surveillance and control strategies because it allows key processes driving infection to be targeted.

Avian influenza viruses (AIV) are multi‐host, viral infections with a global distribution and a complex, multi‐scale transmission ecology (Olsen et al. 2006). AIV in wild waterfowl threaten the domestic poultry industry and potentially play a role in the emergence of human influenza. Single, low pathogenic outbreak losses in domestic poultry are estimated minimally at $131 million (Capua and Alexander 2004) and high pathogenic outbreak losses are estimated at more than $1.15 billion for response/indemnity costs and up to 3.3 billion when accounting for the economic impacts of lost trade (U.S. Department of Agriculture 2016). Predicting the spatiotemporal distribution of AIV across the United States is an important component of broader data‐ and model‐driven management frameworks for wildlife disease (Miller and Pepin et al. 2019) and could improve our understanding of infection ecology (Hill and Runstadler 2016). Previous studies characterizing the distribution of AIV have relied on host traits or local‐scale predictors that capture infection patterns among birds aggregated at one location (Ip et al. 2008, Farnsworth et al. 2012, Bevins et al. 2014, Belkhiria et al. 2016, Papp et al. 2017). Given the broad distribution of wild bird reservoirs and mounting genetic evidence that long‐distance bird movements contribute to the dispersal of AIV (theoretical [Brown et al. 2013, Lisovski et al. 2018]; viral dispersal [Tian et al. 2015, Hill et al. 2016, Toor et al. 2018]), fully understanding AIV spread and distribution also requires assessment of processes acting at larger scales through long‐distance bird movements. For example, although it is well established that AIV prevalence is highest at fall staging areas and decreases over the season as birds migrate south, it remains unknown how long‐distance movements interact with local‐scale processes to influence this pattern.

Hypotheses regarding processes that impact the spatiotemporal distribution of AIV can be categorized into three conceptual areas that vary with respect to mechanism and the spatial scale at which they operate. First, at the scale of individuals, hosts may vary in susceptibility or exposure due to age, sex, and breeding status. AIV prevalence is higher in young birds (Farnsworth et al. 2012, Papp et al. 2017) and usually higher males, but not always (Wallensten et al. 2007). Young, immunologically naïve birds shed a higher amount of virus due to both age‐specific susceptibility (Costa et al. 2010) and the lack of humoral immunity (Jourdain et al. 2010, Dannemiller et al. 2017) such that differences in the spatial and temporal distribution of demographic classes may impact the distribution of AIV. Second, at the local scale, fecal/oral transmission among birds aggregated in one location and transmission from environmental reservoirs over longer timescales (VanDalen et al. 2010) influences infection dynamics (Breban et al. 2009, Fuller et al. 2010, Farnsworth et al. 2012, Belkhiria et al. 2016, Papp et al. 2017). Birds shed virus in fecal material deposited in water and both temperature and variability in temperature impact AIV viability in water (Brown et al. 2009, Keeler et al. 2014). Thus, waterfowl in some habitats have higher AIV prevalence due to differences in the accumulation and persistence of virus in the environment (Fuller et al. 2010, Belkhiria et al. 2016). Third, the contact and mixing patterns among wild birds at local, intermediate, and continental scales generate a contact network that may influence the spatial and temporal distribution of AIV. For example, bird movement into an area, the migration of birds within biological flyways, and continental‐scale mixing among flyways may all mediate infection by influencing transmission among aggregated birds and virus introduction (Hill et al. 2012, 2016, Tian et al. 2015, Sullivan et al. 2018). Few studies have monitored waterfowl movement beyond pairwise connections in order to relate the dynamic network generated through migration to AIV dynamics.

In this work, we integrate across scales and processes to determine the relative importance of different hypothesized mechanisms controlling the continental‐scale distribution of infection. We evaluate whether the long‐distance, intermediate and continental‐scale movements of waterfowl (Hoye et al. 2011, Hill et al. 2012, Tian et al. 2015) or smaller‐scale processes (host aggregation, viral persistence, demography) have relatively more explanatory power. To evaluate multiple spatial scales of waterfowl movements, we develop a novel application of network theory to bird banding/recovery data to create a contact network of migratory waterfowl. We combine this network with an extensive data set on low pathogenic, Type A AIV in migratory waterfowl for the United States (developed as part of the U.S. Interagency Strategic Plan and characterized in DeLiberto et al. [2009], Farnsworth et al. [2012], Bevins et al. [2014]). Our approach uses covariates associated with each hypothesis in a model selection framework to test their relative ability to predict the probability of any AIV infection in individual birds across space and time. We confirm the validity of the selected model for predicting the spatiotemporal distribution of AIV across the United States using standard approaches for goodness‐of‐fit, cross‐validation, and prediction in a subsequent year. We then discuss the implications of our results for surveillance and risk mitigation of AIV in the United States.

## Material and Methods

### Data

Avian influenza surveillance data were gathered via targeted sampling of wild, migratory birds in all 50 states. AIV surveillance data collection was coordinated by USDA, which included sampling by USDA‐Wildlife Services employees and state and tribal partners from 2006–2011 (U.S. Interagency Working Group 2006, DeLiberto et al. 2009, Pedersen et al. 2010, Bevins et al. 2014). Sampling was targeted to collect samples in priority states each biological year (1 April to the following 31 March; DeLiberto et al. 2009, Bevins et al. 2014), which approximately corresponds to the start of nesting and brood rearing in mallards. The spatial and temporal patterns in sampling effort are, therefore, neither random nor regular, but the sampling design resulted in a broad spatial and temporal coverage (DeLiberto et al. 2009). The timing of sampling in priority locations reflects when birds, staff, and/or hunters were present in priority areas, with 82% of samples collected in the hunting season (September–January) and 18% out of the hunting season (February–August). Here, we use a subset of the data from April 2007–2009 that detects the presence or absence of any AIV in cloacal and oropharyngeal swabs. Additional details on data collection methods are provided in Appendix S1: standardization, stratification, and specific diagnostic methods for the matrix real‐time reverse transcriptase‐polymerase chain reaction (rRT‐PCR) assay.

Bird Banding Laboratory (BBL) data were compiled from 2003–2009 to characterize the spatiotemporal patterns of movement (U.S. Geological Survey Bird Banding Laboratory 2010). We used 53,117 banding and recovery records and restricted our analysis to birds that were banded and recovered within the same season (i.e., within 25 weeks of banding) to build a contact network. BBL data included the date and location for banding and recovery, type of recovery, species, sex, and approximate age at banding. Age was defined based on whether or not birds were known to have hatched in the calendar year, called hatch‐year or after‐hatch‐year birds. Although we recognize the potential for bias in the BBL data (e.g., banding locations were not chosen in a probabilistic manner, while harvest and reporting rates vary geographically), it is the best available information on continental‐scale movement in the western hemisphere (Munro and Kimball 1982). Despite the inherent biases in the BBL data, multiple metrics from the data set correlate with known migration patterns (Buhnerkempe et al. 2016) and were predictive of AIV prevalence in this work.

### Mallards as a surrogate for waterfowl

We focus on Mallards (*Anas platyrhynchos*) in the AIV surveillance and BBL data sets because they are the most abundant, widely distributed waterfowl species, are known to amplify and shed AIV (VanDalen et al. 2010, Costa et al. 2011) and have the potential to interact with domestic poultry (Pepin et al. 2014). As the bulk the AIV surveillance data set are mallards, the 28,925 mallard records result in a sufficiently large sample size for modeling (2007‐2008 data; Appendix S1: Table S1). Previous studies characterizing AIV prevalence across species in this data set (Farnsworth et al. 2012, Bevins et al. 2014) and data sets representing a smaller spatial scale (Fuller et al. 2010, Papp et al. 2017) support this focus: prevalence for dark geese is considerably lower than for the dabblers whereas the prevalence in dabblers closely tracks the overall prevalence across all species (Appendix S1: Table S1, Fig. S1). Appendix S1: Fig. S1 illustrates the marginal distributions for AIV prevalence observed in all species, dabbling ducks and dark geese, respectively.

### Network construction

We generated a contact network describing mallard migration (Buhnerkempe et al. 2016) using BBL data and analysed it at multiple scales using metrics derived from network theory (Newman 2010). Specifically, we defined the network by specifying nodes as spatial locations on a 200 × 200 km lattice grid. Edges in the network were defined as the number of mallards moving between two nodes at a four‐week (monthly) time resolution averaged across 5 yr. For example, the edge weights assigned for the first four‐weeks in 2007 were calculated by (1) compiling recovery records for those weeks in 2003, 2004, 2005, 2006, and 2007; (2) quantifying the number of records describing movement along edges in each year; and (3) assigning edge weights as the average number across years. We use this edge definition to align the BBL and AIV surveillance data sets because recovery distributions did not differ significantly across years (Mielke and Berry 2007) and averaging across years minimizes noise due to unobservable bird movements or variation in hunting effort (Roy et al. 2015). Extended network methods and a justification of these spatial and temporal decisions are provided in Appendix S2.

### Data analysis: translating hypotheses into model covariates

**Fig. 1:**
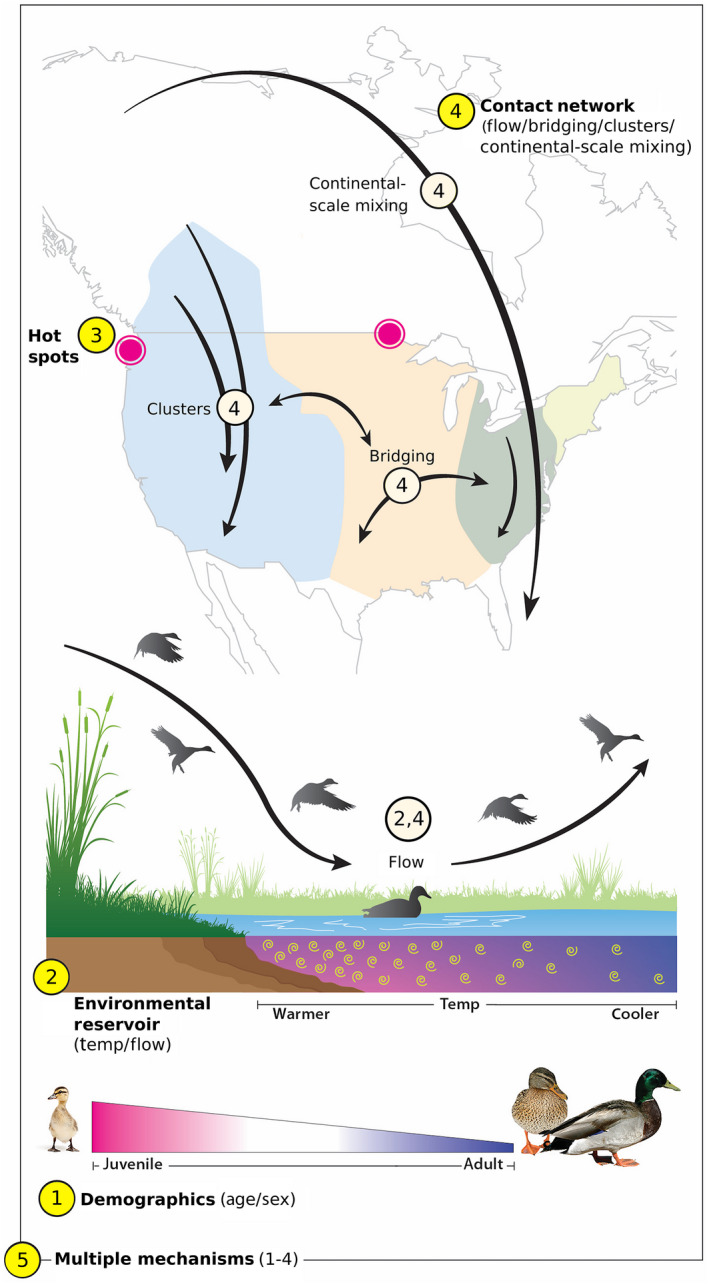
Conceptual diagram of the five hypothesized mechanisms controlling the spatial‐temporal distribution of avian influenza viruses (AIV). (1) Demography hypothesis: young, immunologically naïve male birds exhibit elevated AIV prevalence. (2) Environmental reservoir hypothesis: local aggregation of hosts across the migratory cycle (flow) and water temperature (temp) determine the size of the environmental AIV reservoir and AIV prevalence. (3) Hot‐spots hypothesis: some areas (pink circles) naturally maintain higher AIV prevalence. (4) Contact network hypothesis: Flow, clusters connected by similar migration patterns (colored areas), mixing occurring among clusters (bridging), and continental‐scale migration all impact AIV prevalence (C2). (5) Multiple‐mechanisms hypotheses. Boldface text and yellow circles align with the five hypotheses in Table 1. Light‐yellow circles indicate sub‐components of the contact network hypotheses.

We characterized multiple hypothesized processes driving the spatial and temporal distribution of AIV and developed sets of covariates representing each hypothesis and combinations of hypotheses (Fig 1; Appendix S3).

1. *The demography hypothesis:* In North America, young birds hatch in the summer on northern breeding grounds. They are exposed and develop immunity to infection on the breeding grounds and in the fall as they migrate to southern over‐wintering areas (Hill et al. 2012). The *demography hypothesis* represents the idea that because young, male birds are more likely to be infected (Costa et al. 2011, Farnsworth et al. 2012, Papp et al. 2017), differences in the spatial and temporal distribution of demographic classes impact the distribution of AIV (Fig. 1). Potential predictors representing this individual‐scale hypothesis are age and sex (Table 1).
2. *The environmental reservoir hypothesis*: At the local scale, the *environmental reservoir hypothesis* represents the prediction that differences in the amount of virus in the environment are influenced by the environmental conditions driving viral persistence; the seasonal aggregation of young, immunologically naïve birds via migration that become infected and shed the virus; and the proportion of older, immune birds (Fig. 1). We represent the age‐specific, seasonal aggregation of migratory birds with flow as a predictor. We calculated banding and recovery flow as the age‐specific (hatch‐year, after‐hatch‐year) weighted in‐degree and out‐degree, standardized by the maximum across all nodes and time periods. Weighted in‐ and out‐degree measure the age‐specific number of banded and recovered birds at a particular node during that month. Because environmental persistence is regulated by the abiotic characteristics of the water, we used a temperature polynomial as a predictor for this hypothesis. We use a larger time scale for the bird movement data than the temperature data because the monthly time resolution averages over mismatches from approximating movement with the BBL data set (see detailed network methods in Appendix S2). The temperature polynomial includes the weekly average minimum temperature and the change in weekly average minimum temperature from the previous week (Table 1).
3. *The hot‐spots hypothesis:* Previous studies have indicated that spatial and temporal “hot spots” of AIV occur: AIV prevalence is higher at northern latitudes and during the late summer months (Bevins et al. 2014). Thus, certain spatial or temporal locations could have inherently higher probability of AIV infection, e.g., a flu season. To investigate this, the *hot‐spots hypothesis* is a phenomenological representation of local processes (Fig. 1), with latitude, longitude, and a polynomial week term as predictors. A polynomial temperature term was also considered as predictors since temperature can also generate a hot‐spots‐like pattern (Reperant et al. 2010). (Table 1).
4. *The contact network hypotheses:* We assess the relative contribution of mechanisms at multiple biologically relevant scales based on networks defined by mallard movement. We considered local (*flow hypothesis*), intermediate (*clusters hypothesis, bridging hypothesis*) and continental scales (*continental‐scale mixing hypotheses*) in the contact network as well as their combined effect with the *contact network hypothesis* (Fig. 1; Table 1).

**Table 1:** Potential predictors considered for each hypothesis.

Hypothesis	Predictors
(1) Demography	age, sex
(2) Environmental reservoir	flow × temp polynomial
(3) Hot spots	latitude × longitude × week polynomial, latitude × longitude × temp polynomial
(4) Contact network	flow, cluster identification, bridging index, minimum spanning trees index (MST)
(5) Multiple mechanisms	age, sex, flow × temp polynomial, latitude × longitude × temp polynomial, cluster identification, bridging index, MST

Notes

Appropriate main effects and lower‐level interactions were included where needed (e.g., a model with Latitude × Longitude × Week polynomial would also include Latitude × Week polynomial, Longitude × Week polynomial). Week polynomial refers to three parameters: week + week^2^ + week^3^. Temp polynomial refers to six parameters: *T*_0_ + $T_{0}^{2}$ + $T_{0}^{3}$ + Δ*T* + Δ*T* ^2^ + Δ*T* ^3^, where *T*_0_ represents the weekly average minimum temperature and represents the change in weekly average minimum temperature from the previous week. Flow is represented by four parameters describing the banding flow for hatch year birds, banding flow for after‐hatch‐year birds, recovery flow for hatch‐year birds, and recovery flow for after‐hatch‐year birds. Hypothesis numbers refer to their display in Fig. 1 and presentation order in *Methods*.

At the smallest, relatively local scale, the *flow hypothesis* predicts that the age‐specific aggregation of migratory birds in a node influences the distribution of AIV, with flow as a predictor. The influx of immunologically naïve migratory birds, plays a dominant role in infection dynamics within a site (Brown et al. 2013, Lisovski et al. 2018).

At the intermediate scale, the *clusters hypothesis* predicts that the migration of birds within biological flyways influences the distribution of AIV. We characterize data‐driven flyways based on groups of highly connected nodes using an algorithm that clusters nodes using connectivity data, a community detection algorithm. We considered clusters as static based on all available data from 2003 to 2008, and we use cluster identity as a predictor representing this hypothesis. A detailed description of the algorithm is provided in Appendix S2. We applied this algorithm because it is appropriate for weighted, directed networks (e.g., it accounts for the direction and strength of the connections) and does not require that the number of clusters be predetermined. Previous analyses of bird movements based on the BBL data using other community detection algorithms produced qualitatively similar flyway identifications (Buhnerkempe et al. 2016).

The *bridging hypothesis*, also at the intermediate scale, predicts that areas where mixing between flyways occurs will mediate infection patterns. The bridging hypothesis is represented with a bridging index that quantifies if a node has connections to multiple clusters, signaling mixing. We defined and calculated a bridging index for each node as the proportion of birds moving through a node that remained within the cluster. Specifically, we calculated the ratio of the bandings/recoveries at a node that moved to/from a node in a different cluster to the total number of bandings/recoveries associated with the node. A value close to one indicates a large amount of mixing with other clusters; a value close to zero implies that the node mixes largely within its own cluster. This statistic is conceptually related to measures of bridging between individuals developed in the social sciences (Valente and Fujimoto 2010). Year‐to‐year variation was estimated by computing the bridging index using only the most recent five years of banding and recovery data (e.g., 2003–2007 data were used to compute bridging for 2007).

To represent the influence of continental scale network structure (*continental‐scale mixing hypothesis*), we used the minimum spanning trees (MST) index. An MST is roughly defined as the smallest set of edges in the network that together connect all the nodes of the network. Hence, the MST provides the shortest path through the network that still maintains 100% connectivity. It is possible to have multiple minimum spanning trees or uncertainty in the minimum spanning tree as in our case where the network is sampled and not fully known. The MST index was calculated for each node; it conceptually represents how important that node is in maintaining a fully connected network. We calculated it with a multi‐step procedure. First, we built a master table of banding to recovery locations such that the connections between nodes were collapsed across time. The resulting table is *n* × *n* and sparse, and we set the main diagonal to zero to account for self‐connections. Second, we used an algorithm to identify subsets of the network that preserve 100% connectivity. The algorithm proceeds by randomly selecting a banding node and checking to see if 100% connectivity is maintained if that node were omitted from the network. This procedure repeats until no nodes remain that can be removed without maintaining 100% connectivity. The set of remaining nodes makes up one subset of the network whose edges form an MST. Because the overall structure of the network is unknown (i.e., we only observed a sample of the connections between nodes across time), we repeated the algorithm to identify 100 subsets. Third, we calculated the MST index for each node as the proportion of subsets that included that node. Year‐to‐year variation was computed using the most recent five years of banding and recovery data similar to the bridging index.

### Data analysis and model selection

We used logistic regression to model the probability that an individual bird is AIV positive or negative. Assumptions of logistic regression were tested and met (e.g., significant spatial or temporal correlation in model residuals was not observed based on spatial plots of the residuals and autocorrelation functions). Model selection proceeded by identifying a candidate set of models that included covariates appropriate to test each hypothesis under consideration (Table 1). Due to the large number of potential models, we conducted model selection in two stages. First, we selected a parsimonious model representing each hypothesis using model selection. In all models, we estimated model parameters and their corresponding standard errors using likelihood‐based methods and compared among models using small‐sample size corrected Akaike’s information criterion (AIC_c_). We selected the model with the lowest AIC_c_ within each hypothesis (Appendix S3: Table S1). Second, the covariates from the selected model for each individual hypothesis were combined to generate a full set of multiple‐mechanisms models to test if multiple types of processes impact the distribution of AIV. We selected the multiple‐mechanisms model with the lowest AIC_c_ and used the change in AIC_c_ values, ΔAIC_c_, to compare the relative contribution of processes, represented by multiple covariates (e.g., environmental reservoir) to the selected model for each individual hypothesis.

We evaluated the model with the overall lowest AIC_c_ value using both in‐sample and out‐of‐sample validation. For in‐sample validation, we assessed goodness of fit by estimating the Receiver Operating Characteristic curve and the area under the curve (AUC; AUC values over 0.5 indicate that the model is able to predict AIV+ vs. AIV− and AUC values over 0.7 indicate good prediction). We also performed cross‐validation to evaluate the stability of the estimated model parameters and to evaluate the overall predictive capability (Arlot and Celisse 2010). For out‐of‐sample validation, we applied the best model with parameter estimates from the 2007 to 2008 data to response and covariate data collected for the 2009 biological year. We tested out‐of‐sample prediction using AUC to quantify the model’s ability to predict AIV+ vs. AIV− for the 2009 biological year. All analyses were conducted in R (R Core Team 2012); network statistics were calculated with custom scripts.

## Results

### Bird movement networks

**Fig. 2:**
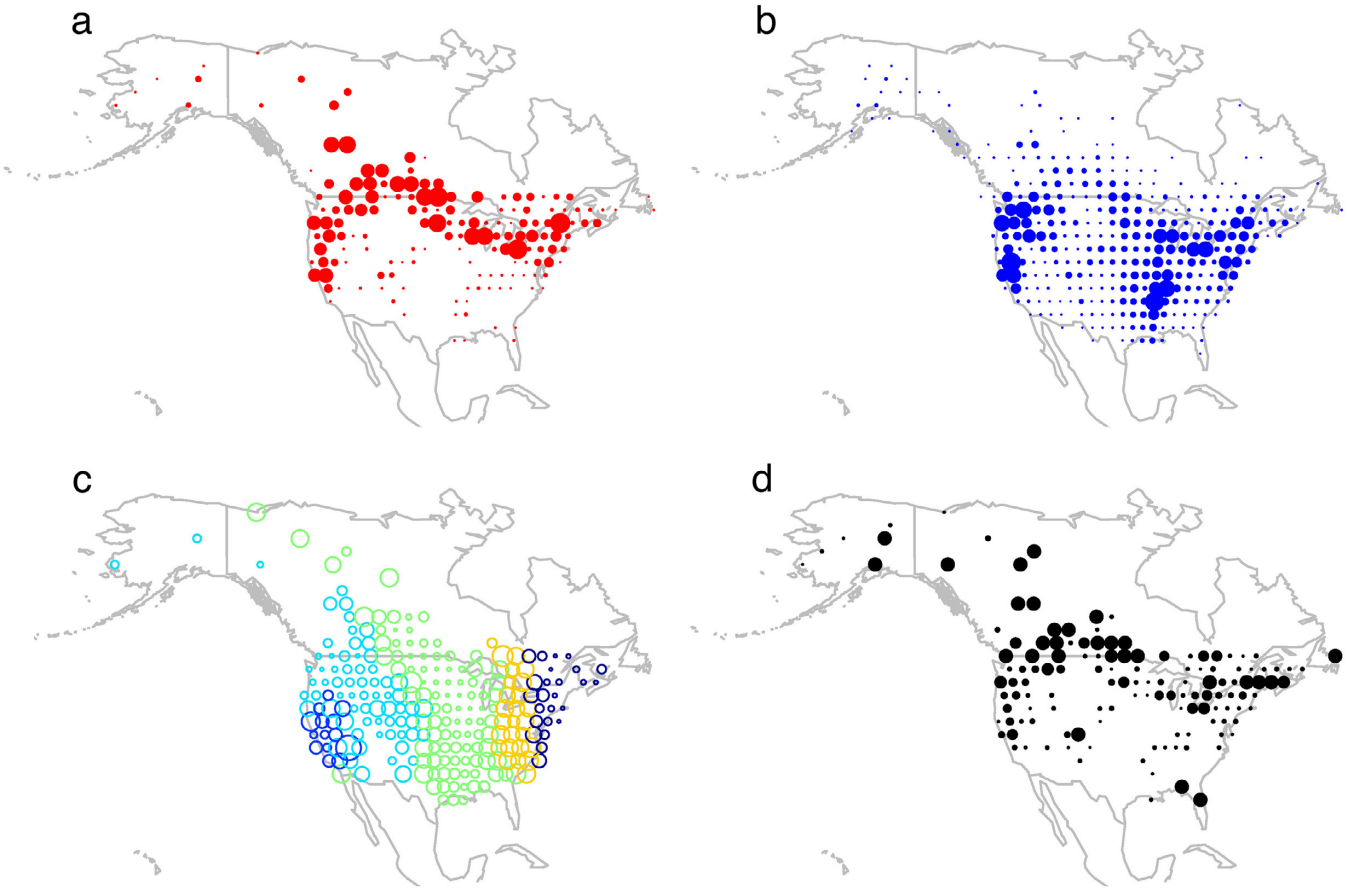
Contact network of bird movements. The circle area is proportional to (a) early‐season banding flow aggregated temporally, (b) late‐season recovery flow aggregated temporally, (c) the bridging index, and (d) continental‐scale mixing based on the minimum spanning tree index. Colors in panel c indicate clusters in the network.

Flow captured changes in the number of birds at each node through time. Fig. 2a shows flow from banding records aggregated to an annual scale. Most banding happens early in the season while birds are still on the northern breeding grounds and west coast in significant numbers. Video S1 illustrates how flow from banding records develops over the year and captures the early season aggregation of migratory of birds (Appendix S3). Fig. 2b shows flow from recovery records aggregated to an annual scale. At the annual scale, we see that aggregation is highest on the West Coast and in the Mississippi River and Great Lakes regions. Video S2 illustrates how flow from recovery records develops over the year as birds migrate south and captures later season aggregation of birds. Aggregation appears to occur earlier in the Mississippi River and Great Lakes regions, yet it is maintained for a longer period of time once it occurs on the West Coast. It moves from north to south along the Mississippi River region.

Fig. 2c shows clusters in the contact network; birds located in one cluster are more likely to migrate to another location within that cluster. Cluster 1 (dark blue) is located in California; cluster 2 (light blue) is in the west and split to either side of the Rocky Mountains; cluster 3 (green) is in the Mississippi valley; cluster 4 (yellow) is in the Ohio valley; and cluster 5 (black) extends along the East Coast. The bridging index tends to be highest on the boundary of clusters, suggesting that cluster boundaries are porous and mixing among clusters occurs primarily at their boundaries. Clusters 1 and 4 have high levels of bridging throughout the cluster. Cluster 1 in California mixes strongly with the western portion of cluster 2. Cluster 4 appears to be a transition zone between the Mississippi valley (cluster 3) and the East Coast (cluster 5). The cluster locations and distribution of the bridging index are fairly consistent with ecological studies on flyways (Buhnerkempe et al. 2016).

Fig. 2d shows locations that play an important role in continental‐scale connectivity due to their inclusion in the MST. Many key locations occur at northern latitudes that control connections to the contiguous United States. Additional important locations occur in Florida and Colorado. Much of the Midwest, Southwest and Southeast are not part of the MST, suggesting that these areas have many connections or routes by which they can be reached through the network and are not key areas in controlling overall connectivity across North America.

### AIV prevalence and model inference

**Fig. 3:**
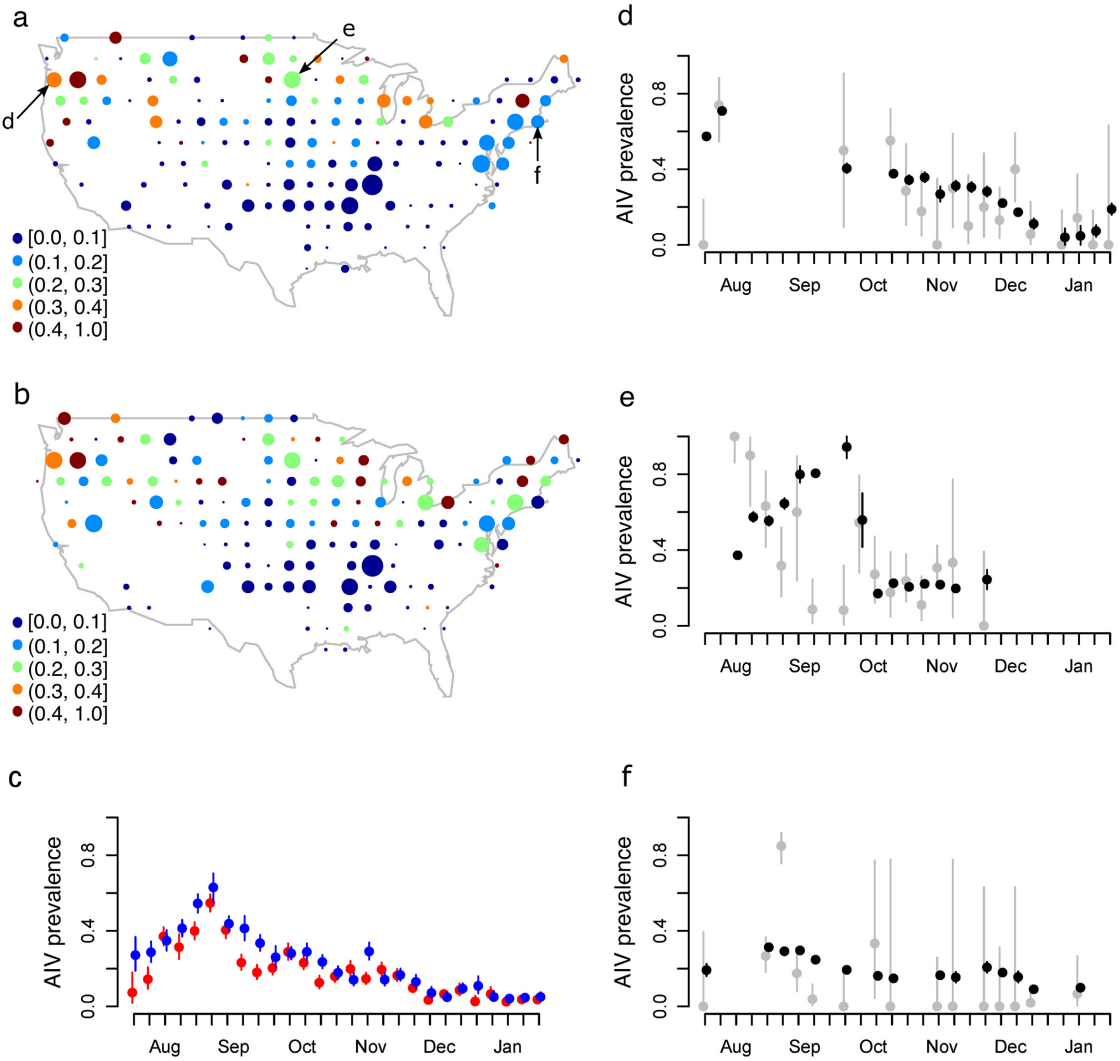
AIV prevalence. Observed AIV prevalence in Mallards at spatial locations across the continental United States aggregated in the (a) 2007 biological year and (b) 2008 biological year. Sample size is represented by circle area. (c) Observed AIV prevalence in Mallards through time, aggregated across the continental United States for 2007 (red) and 2008 (blue). The symbols show the observed mean prevalence and lines indicate 95% confidence intervals (Appendix S2). (d–f) Comparison between observed data (light gray) and model predictions (black) across time at three distinct nodes. Node locations correspond to letters in (a), chosen to display a range of spatial locations and prevalence values.

The highest AIV prevalence occurred in northern latitudes, particularly in the Pacific Northwest, Great Lakes, and Northeast (Fig. 3a, b) and in the mid‐ to late‐summer months (Fig. 3c). While it is easier to describe and visualize the AIV distribution at the continental scale with prevalence, our model predicts the disaggregated information underlying prevalence: the AIV infection status of individual birds. A combination of hypotheses represented in the multiple‐mechanisms model was best supported by the data set (Table 2). The selected, best‐fit multiple‐mechanisms model resulted in a much lower AIC_c_ value compared to each individual hypothesis model (Table 2; Appendix S3: Table S1). The multiple‐mechanisms model also resulted in a good fit to the overall spatiotemporal distribution of AIV in 2007 and 2008 as determined by individual bird AIV infection status (AUC = 0.76), although prevalence at some specific locations and times visually appear over or under predicted (Fig. 3d–f). The multiple‐mechanisms model had substantial predictive ability under cross‐validation, as suggested by similar values for error sum of squares and pure error sum of squares (within 0.9%; Appendix S3: Fig. S1). It also performed well during out‐of‐sample validation (AUC = 0.69) based on predicted individual infection status using 2007–2008 parameter estimates and 2009 covariate data as compared to the observed infection status of 7,108 mallards sampled in the 2009 biological year. Again, prevalence at some locations and times visually appear over or under predicted (Appendix S3: Fig. S2).

**Table 2:** Model selection and relative contribution of predictors in the selected model.

Model	Contact network hypothesis	Environmental reservoir hypothesis	*p*	p*	AIC_c_	AUC	ΔAIC_c_
Multiple mechanisms			64		18,559	0.76	
Hot spots			28		19,371	0.74	
Environmental reservoir			35		19,290	0.70	
Contact network			13		19,874	0.70	
Demography			3		21,114	0.63	
Subsets of multiple‐mechanisms model							
Age				1	18,722		163
Bridging and cluster	x			7	18,585		26
Flow	x	x		4	18,563		4
Temp polynomial		x		6	18,607		48
Flow × temp polynomial		x		24	18,693		134

Notes

The model selection table includes the numbers of parameters (*p*), corrected Akaike information criterion (AIC_c_) and area under the Receiver Operating Characteristic curve (AUC). Lower AIC_c_ values indicate greater support, AUC values over 0.5 indicate that the model is able to discern avian influenza virus (AIV)+ vs. AIV−; values over 0.7 indicates good discernment; values of 1 indicate that the model is always correct. Comparing subsets of the multiple‐mechanisms model, the number of parameters (*p* ^*^) are those associated with biologically interpretable groups of predictors and the ΔAIC_c_ measures the relative contribution associated with including those terms in the multiple‐mechanisms model.

**Fig. 4:**
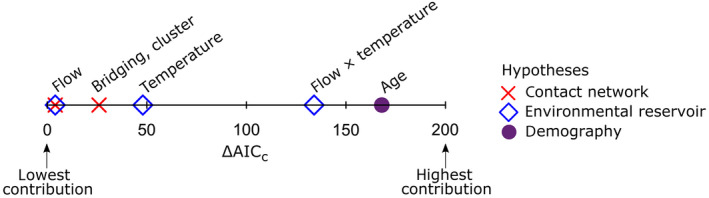
Relative contribution of predictors in the final, multiple‐mechanisms model based on the change in the Akaike information criterion corrected for sample size (ΔAIC_c_). Larger ΔAIC_c_ values indicate that those predictors have a larger contribution to the multiple‐mechanisms model. Parameters are combined into biologically interpretable groups of predictors and colored based on the hypothesis they represent (Table 1). For example, flow is represented by four parameters describing the banding flow for hatch year birds, banding flow for after‐hatch‐year birds, recovery flow for hatch year birds and recovery flow for after‐hatch‐year birds. Flow is a predictor in both the contact network and environmental reservoir hypotheses.

To better understand the relative role of different processes and spatial scales, we interpreted the relative contribution made by groups of parameters associated with specific hypotheses to the multiple‐mechanisms model (Fig. 4), as well as parameter estimates that relate individual predictors to the probability of AIV in individual birds (Appendix S3: Table S2).

An important role for the environmental reservoir was supported by the highest sum ΔAIC_c_ values for the associated covariates: flow, temperature, and their interaction (Table 2; Fig. 4). Significant parameter estimates and the relatively high ΔAIC value for the flow by temperature interaction terms supported an important role of environmental reservoir transmission as differentiated from direct, fecal/oral transmission mechanisms alone (e.g., potentially associated with flow covariates) or temperature only effects (e.g., associated with the temperature polynomial; Fig. 4). The role of demography was also supported as the age covariate had the next highest ΔAIC_c_ value. Consistently, the probability of positive AIV status decreased with age (after hatch year compared to hatch year; β = 0.057, *P* < 0.001). Finally, the bridging and cluster covariates are included in the best‐fit model, providing some support for a potential role of higher order network‐based features of the migration process. Despite clear latitudinal and temporal patterns in the distribution of AIV, there was no direct evidence for a main latitudinal or temporal effect after accounting for demographic, environmental, and bird‐movement based predictors. Parameter values for latitude and longitude, as well as their interaction, were small and non‐significant (latitude, β = −0.04, *P* = 0.740; longitude, β = 0.10, *P* = 0.059; interaction, β = −0.001, *P* = 0.163; Appendix S3: Table S2), and the week polynomial was not selected for inclusion in the final model. However, there were some higher order interaction terms of latitude and longitude with temperature in the model; we speculate on some alternative roles for these terms in the discussion.

## Discussion

For wildlife disease systems, multiple ecological mechanisms often contribute to the distribution of infection and risk of spill‐over infection in domestic animals (e.g., bTB [Palmer et al. 2012], Nipah virus [Pulliam et al. 2011], and rabies [Blackwood et al. 2013]). In this study, we illustrate how synthesizing data across multiple scales of ecological organization into a hypothesis‐testing framework can identify the relative contribution of these processes. Using AIV infection in migratory waterfowl, we show that continental‐scale infection patterns may be influenced by processes acting at multiple scales.

Our findings provide novel insights into the drivers of AIV infection in wild waterfowl. The greater contribution of individual and relatively local spatial‐scale processes in our analysis (Fig. 4) suggests that processes acting at these levels predominate and scale up to influence the distribution of AIV at the continental scale. Specifically, our results support hypotheses representing local, environmental reservoirs and demography (age) as driving the probability of infection and ultimately prevalence. Here, age and age‐specific aggregations of migratory birds approximate immune status and population immunity, respectively. Previous work has experimentally characterized the age‐specific (Costa et al. 2010) and environmental drivers of infection (Brown et al. 2009, Keeler et al. 2014). Studies integrating detailed data collection and theoretical models based on one location have demonstrated the importance of environmental reservoirs, the role of population immunity and the influx of young, immunologically naïve individuals through births and migration (Breban et al. 2009, Brown et al. 2013). For example, detailed sampling and modeling at one site in Oud Alblas, Netherlands, suggest that the influx and replacement of young, immunologically naïve migratory birds are required to predict the local dynamics of infection at one location (Lisovski et al. 2018). The overall support for the environmental reservoir hypothesis (Fig. 4), capturing interactions between temperature and local, age‐specific aggregations of birds across the migratory cycle, indicates a broader role of this mechanism at the continental scale.

Our work also quantifies the movement of birds in a network context and provides a data‐driven assessment of multiple scales of the migration process. Most work on the role of bird movements in the spread of AIV has focused on the inferred movement of birds between two to a few locations (Hoye et al. 2011, Hill et al. 2012, Hill et al. 2012, Hill et al. 2016) or the role of migratory flyways (Fourment et al. 2017; but not always, Tian et al. 2015, Sullivan et al. 2018). Migratory flyways represent common migratory paths and are presumed to represent populations that can be discretely managed (Lincoln 1935). They have been shown to influence viral movement by restricting transmission between flyways (Fourment et al. 2017). Our work suggests that flyways, represented here by data‐driven clusters, had less influence on AIV prevalence compared to temperature and migratory bird movement quantified as aggregation at the local scale (Table 1; relative support for flow vs. clusters hypothesis). This result underscores the difference between strain‐specific viral movement and prevalence. Different processes likely influence viral movement vs. prevalence because the latter is dependent on both the virus being moved into the area and the following local dynamics (Lisovski et al. 2018). Additionally, most studies of viral movement are based on canonical north‐south flyways (Fourment et al. 2017) that may miss species‐specific differences, seasonal differences, or local movements that are revealed using banding data (La Sorte et al. 2014, Buhnerkempe et al. 2016). For example, the high levels of bridging in the California and Ohio valley clusters as well as at the boundaries of flyways suggest that these clusters and their boundaries may not be as static as the others. Understanding how locations with high levels of connectivity or bridging influence transmission will be an important area of future research. Such a research agenda will be able to leverage a well‐developed theory of disease transmission on contact networks (Keeling 2005).

Furthermore, our work suggests that these mechanisms explain more variation in AIV prevalence compared to a more phenomenological representation of spatial and temporal hot spots represented in the hot‐spots model (AICc values in Table 1). The hot‐spots model resulted in good prediction based on AUC values, consistent with previous work establishing its predictors (latitude, longitude, week) as correlates of AIV prevalence (Ip et al. 2008, Farnsworth et al. 2012, Brown et al. 2013, Bevins et al. 2014). However, these terms had a low contribution to the final model and were non‐significant after accounting for local, age‐specific bird movements and temperature. Some complexity still remains in the multiple‐mechanisms model from higher order latitude, longitude and temperature interactions (Appendix S3: Table S2). These terms may correct mismatches between the data sets resulting from approximating migration from the BBL data, which is influenced by variable harvest and reporting rates (Munro and Kimball 1982). They may also represent unobserved host or virus‐specific predictors of AIV. Specifically, by focusing on local, intermediate and continental‐scale mechanisms, our work does not consider variation within or between subtypes (Latorre‐Margalef et al. 2014), co‐infections (Wille et al. 2015), or heterogeneity among habitats within a location (Sullivan et al. 2018).

Surveillance and risk mitigation for spillover between wild birds and domestic poultry is complex and worthy of data and modeling frameworks that consider how these smaller‐scale mechanisms combine with the local, intermediate, and continental‐scale movement of birds (Pepin et al. 2014, Ramsey et al. 2014). Therefore, a current challenge for surveillance systems in North American wild birds is to balance multiple objectives; surveillance goals include (1) maximizing early detection of novel strains of introduced, highly pathogenic AIV, (2) identifying regions that would receive the largest benefits from the establishment of risk mitigations, and (3) understanding the dynamics of commonly circulating low pathogenic AIV, including the spatial‐temporal drivers of prevalence, subtype diversity, and reassortment (Bevins et al. 2014, 2016). Our results suggest that the Pacific Northwest and Great Lakes regions have a high AIV prevalence in wild waterfowl. This area also has frequent backyard poultry operations or live bird markets; it also has high densities of small, higher‐risk commercial poultry operations (Appendix S1: Fig. S1). Given the potential introduction of commonly circulating AIVs and subsequent emergence of high path phenotypes in domestic poultry (Xu et al. 2017, Li et al. 2018), this work highlights the importance of continued monitoring. Additionally, the importance of processes acting at local scales identified here suggests that some aspects of surveillance and risk assessment for endemic AIV could be focused at these scales. Emphasis could be placed on assessing the local density of hatch‐year and after‐hatch‐year birds and on water temperatures as opposed to more logistically complex tracking of bird migration patterns or even regional movements from areas of higher AIV prevalence. Coordination of resource distribution and data analysis for AIV continues to be needed at a national scale, particularly because changes in smaller‐scale processes due to climate change and other factors will be difficult to track otherwise.

## Supporting information

Appendix S1

Appendix S2

Appendix S3

Video S1

Video S2

Video S1Legend

Video S2Legend

## Data Availability

Bird banding, recovery, and metadata are available from the USGS Bird Banding Laboratory (https://www.usgs.gov/centers/pwrc/science/bird‐banding‐laboratory). To obtain the data, follow the “Data and Tools” link, then the “Request data from the BBL” link. This data set included mallard recovery records from 2003–2009. The avian influenza surveillance data are stored, maintained, and available upon request from the National Wildlife Disease program at USDA's National Wildlife Research Center (see up‐to‐date contact details here: https://www.aphis.usda.gov/aphis/ourfocus/wildlifedamage/programs/nwrc/nwdp). This data set included samples from mallards from 2007 to 2009.
