# Supplementary material for: Continental‐scale dynamics of avian influenza in U.S. waterfowl are driven by demography, migration, and temperature
Source: Ecol Appl. 2020 Nov 22;31(2):e2245. doi: 10.1002/eap.2245 (PMC7988533; doi:10.1002/eap.2245)
Supplement: Supplementary file 1 — Appendix S1 [file EAP-31-e2245-s006.pdf]

Supporting Information. Gorsich, E.E., C.T. Webb, A.A. Merton, J.A. Hoeting, R.S. Miller, M.L. Farnsworth, S.R. Swafford, T.J. DeLiberto, K. Pedersen, A.B. Franklin, R.G. McLean, K.R. Wilson, and P.F. Doherty, Jr. 2020. Continental-scale dynamics of avian influenza in U.S. waterfowl are driven by demography, migration, and temperature. Ecological Applications.

APPENDIX S1. ADDITIONAL DATA METHODS

Avian influenza surveillance data

Avian influenza surveillance data were gathered using standardized sample collection methods and diagnostic testing procedures in accordance with the U.S. Interagency Strategic Plan. Stratified longitudinal sampling accounted for general migratory patterns at the continental scale. Date of sampling, approximate geographic location, species, sex, and approximate age (hatch-year or after hatch-year) were recorded. In all analyses, we used a subset of data where cloacal and oropharyngeal swabs were collected from apparently healthy birds, placed in Brain Heart Infusion media, and tested at a National Animal Health Laboratory Network facility. Swabs were left in the sample vial and remained defrosted until screened for type A influenza virus with the matrix real-time reverse transcriptase-polymerase chain reaction (rRT-PCR) assay. Additional rRT-PCR testing and virus isolation were performed; however, these data were not used in the analysis. Sample sizes and observed AIV prevalence for major groups and key species in the data set are described in Table S1.

Table S1. Sample size and observed AIV prevalence for major groups and key species in the data set for biological year 2007 and biological year 2008.

**Group/Species**	**Number**		**%AIV positive**	
	**2007**	**2008**	**2007**	**2008**
Dabbling duck (12 species)	39198	40482	12.6	14.2
American green-winged teal (*Anas carolinensis*)	5802	6898	11.6	13.3
Blue-winged teal (*Anas discors*)	3349	3323	15.3	15.3
Mallard	14041	14884	16.4	19.1
Northern pintail (*Anas acuta*)	3228	2893	16.7	16.3
Wood duck (*Aix sponsa*)	3317	3507	3.5	5.8
Dark geese (4 species)	5795	7799	2.5	2.1
Canada goose	5183	6828	2.6	2.3
All Species (119 species)	56497	60138	9.9	11.1

(A)

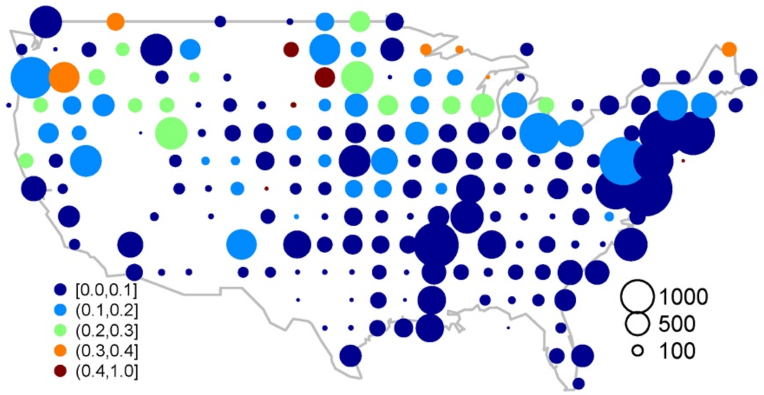
[0.0,0.1]
(0.1,0.2]
(0.2,0.3]
(0.3,0.4]
(0.4,1.0]
1000
500
100

(B)

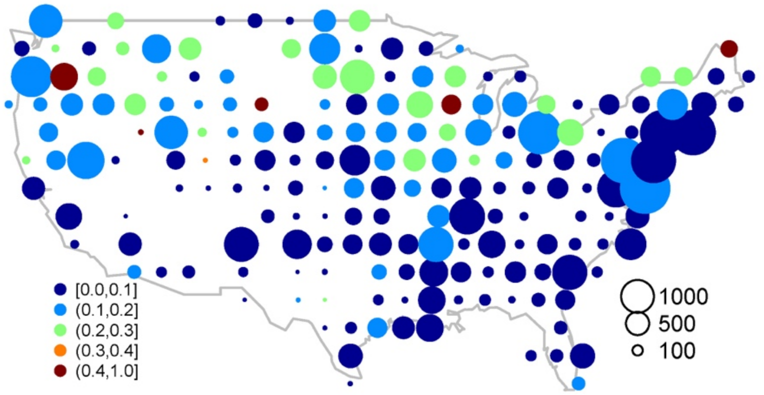
[0.0,0.1]
(0.1,0.2]
(0.2,0.3]
(0.3,0.4]
(0.4,1.0]
1000
500
100

(C)

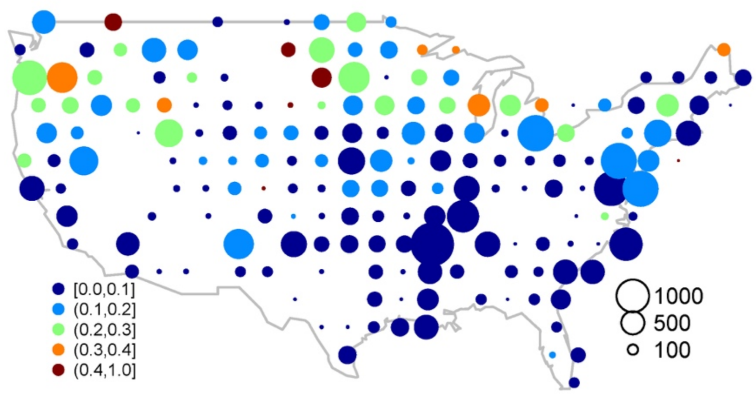
[0.0,0.1]
(0.1,0.2]
(0.2,0.3]
(0.3,0.4]
(0.4,1.0]
1000
500
100

(D)

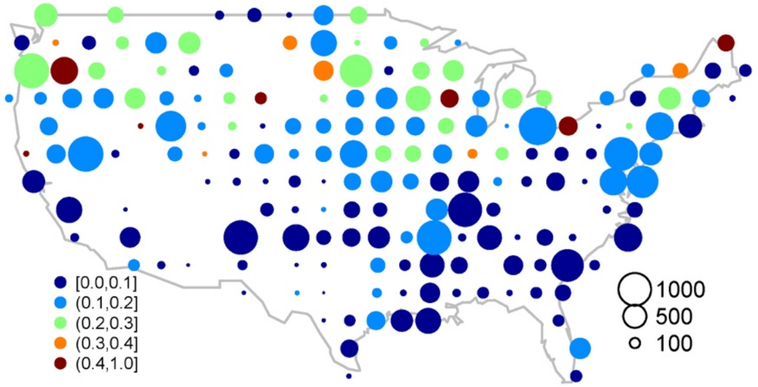
[0.0,0.1]
(0.1,0.2]
(0.2,0.3]
(0.3,0.4]
(0.4,1.0]
1000
500
100

(E)

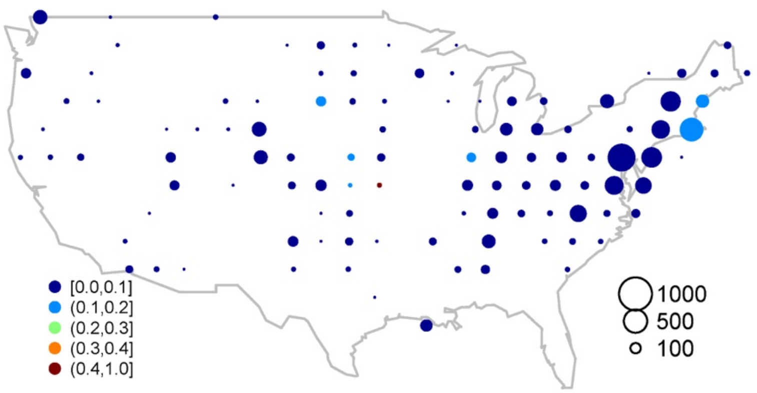

(F)

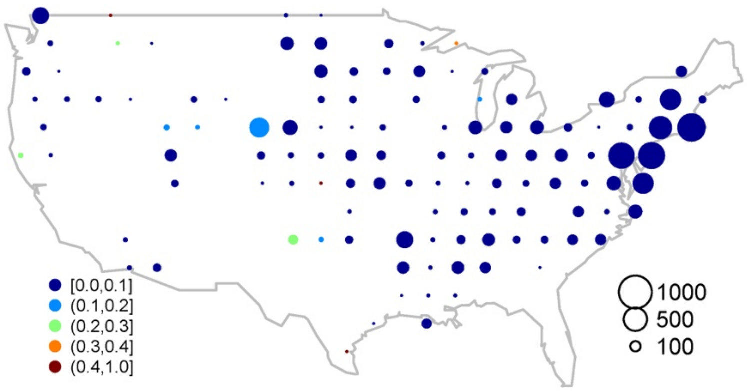

Figure S1. Observed AIV prevalence. Circle area proportional to sample size. (A, B) Spatial distribution of AIV prevalence for all species (119 species) in the AIV surveillance data for the biological years 2007 and 2008, respectively. (C, D) Spatial distribution of AIV prevalence for dabbling duck species (12 species) for the biological years 2007 and 2008, respectively. (E, F) Spatial distribution of AIV prevalence for dark geese (4 species) for the biological years 2007 and 2008, respectively.

Temperature data

Because temperature impacts AIV viability in water [1,2], we used ambient temperature data generated by the Parameter-elevation Regressions on Independent Slopes Model [3] (PRISM) and interpolated as the mid-point between monthly estimates at each node (see network construction in the main text) to compute weekly average minimum temperatures and the change in weekly average minimum temperatures. We assumed that water temperature was correlated with ambient temperature [4]. Water salinity and pH are also known to impact AIV viability, but they have a smaller impact on viral persistence within the ranges observed in freshwater systems [1,2] and these data were not available at appropriate scales for our analyses.

Poultry data

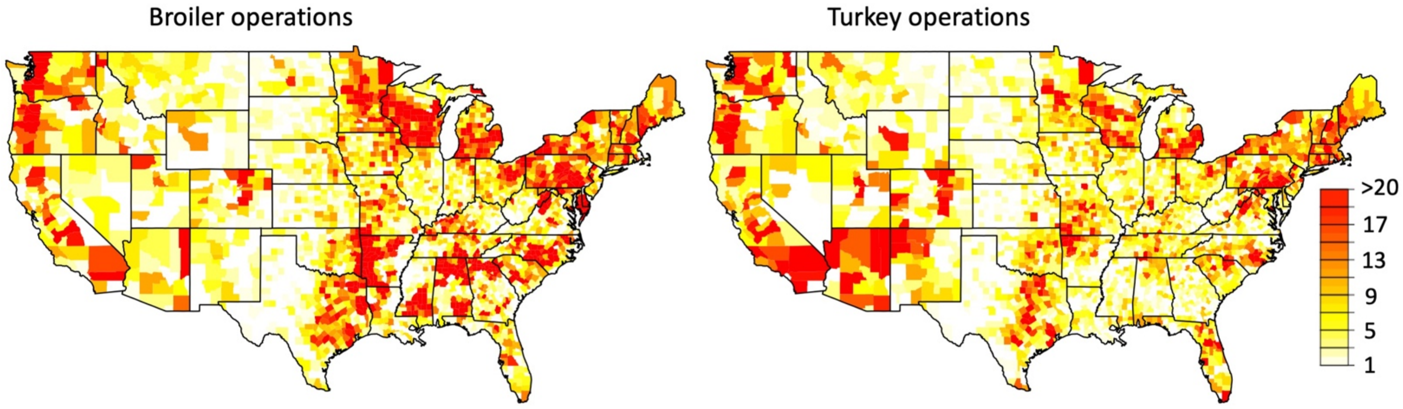

Figure S2. Distribution of poultry operations in the continental U.S. Colors represent the relative number of (a) broiler operations and (b) turkey operations. Data on poultry location records at the county level were obtained from the United States Department of Agriculture National Agricultural Statistics Service survey in 2007 (https://quickstats.nass.usda.gov/).

Additional references

1. Brown JD, Goekjian G, Poulson R, Valeika S, Stallknecht DE. 2009 Avian influenza virus in water: Infectivity is dependent on pH, salinity and temperature. *Vet. Microbiol.* **136**, 20–26. (doi:10.1016/j.vetmic.2008.10.027)

2. Keeler SP, Dalton MS, Cressler AM, Berghaus RD, Stallknecht DE. 2014 Abiotic Factors Affecting the Persistence of Avian Influenza Virus in Surface Waters of Waterfowl Habitats. *Appl. Environ. Microbiol.* **80**, 2910–2917. (doi:10.1128/AEM.03790-13)

3. Daly C, Neilson RP, Phillips DL. 1994 A Statistical-Topographic Model for Mapping Climatological Precipitation over Mountainous Terrain. *J. Appl. Meteorol.* **33**, 140–158. (doi:10.1175/1520-0450(1994)033<0140:ASTMFM>2.0.CO;2)

4. Caissie D. 2006 The thermal regime of rivers: a review. *Freshw. Biol.* **51**, 1389–1406. (doi:10.1111/j.1365-2427.2006.01597.x)
