# Supplementary material for: Continental‐scale dynamics of avian influenza in U.S. waterfowl are driven by demography, migration, and temperature
Source: Ecol Appl. 2020 Nov 22;31(2):e2245. doi: 10.1002/eap.2245 (PMC7988533; doi:10.1002/eap.2245)
Supplement: Supplementary file 2 — Appendix S2 [file EAP-31-e2245-s005.pdf]

Supporting Information. Gorsich, E.E., C.T. Webb, A.A. Merton, J.A. Hoeting, R.S. Miller, M.L. Farnsworth, S.R. Swafford, T.J. DeLiberto, K. Pedersen, A.B. Franklin, R.G. McLean, K.R. Wilson, and P.F. Doherty, Jr. 2020. Continental-scale dynamics of avian influenza in U.S. waterfowl are driven by demography, migration, and temperature. Ecological Applications.

APPENDIX S2. ADDITIONAL STATISTICAL METHODS

Network construction

We defined the network by specifying spatial locations as nodes of the network and specifying connections through bird movements as edges and edge weights of the network. Nodes in the network were defined with a 200 km x 200 km lattice grid with distances calculated using Great Circle Distance to maintain both a constant distance between and constant area across adjacent nodes. A 100 km × 100 km lattice contained too many zero counts in space and time among the BBL data to effectively capture the network metrics. Our spatial locations included Alaska and Canada in addition to the continental U.S. in order to capture movements connected through northern sites. Edges in the network were defined as the average number of birds moving between two nodes at either a weekly or four-week (monthly) time resolution taken across years based on a five-year moving window (e.g., 2003-2007 data as a proxy for the 2007 biological year). We use this edge definition to align the BBL and AIV surveillance datasets for multiple reasons. First, recovery distributions did not differ significantly across years [1].

By averaging across years, we minimize noise due to unobservable bird movements and maximize its representativeness of movement. Second, weekly and monthly time resolutions were selected for the analysis, primarily to smooth variability in BBL recovery counts. Hunter recoveries are more likely to occur on weekends, and the dates and locations associated with large numbers of recoveries tend to shift over the week from year to year. Third, birds typically shed AIV for 5-12 days depending on strain and waterfowl species [2,3], making these time scales biologically relevant. Because weekly and monthly metrics were highly correlated, we used the monthly metric of flow in the models as it was parsimonious.

Community detection

We identified clusters in the network, which are defined as sets of nodes in the network with high levels of connections based on bird movement among them and low levels of connections to other nodes. Clusters were considered static and were identified using all of the available banding and recovery data from the years 2003 through 2008. Our clustering algorithm accounts for the strength of the connections between nodes in a directed network and does not require that the number of clusters be predetermined.

The algorithm proceeds by first assigning each node to a cluster starting with the two nodes with the strongest connection (i.e., the highest number of birds traveling between nodes). These two nodes are assigned to cluster 1. If the second strongest connection links a third node to either of the first two nodes then this third node is also assigned to cluster 1; otherwise the two nodes of the second strongest connection are assigned to cluster 2. The algorithm proceeds in this manner until all of the nodes are assigned.

The algorithm then anneals the initial structure by reassigning the individual nodes that are currently more strongly inter-connected, i.e., connected to more nodes outside its current cluster assignment, to another cluster; the process repeats until all nodes are at least as strongly intra-connected as inter-connected. During this process, clusters may be merged with other clusters to form larger clusters.

Confidence intervals for observed data

To construct the point estimates for observed prevalence (Fig. 3 and Appendix C, Fig. C3), we used the principle of maximum likelihood estimation. Let n be the number of individuals tested and let y_{ijk} be the test result for the ith individual at the jth node in the kth time period (where $y_{ijk} = 1$ indicates positive and $y_{ijk} = 0$ indicates negative). If we assume the probability of a randomly selected individual is positive and equals π_{jk}, then the corresponding likelihood function for π_{jk} given n_{jk} and $\boldsymbol{y}_{jk} = (y_{1jk}, \ldots, y_{njk})'$ is

$$L(\pi_{jk}; n_{jk}, \boldsymbol{y}_{jk}) = \prod_{i=1}^{n_{jk}} \pi_{jk}^{y_{ijk}} (1 - \pi_{jk})^{1-y_{ijk}}.$$

The likelihood function is maximized at $\hat{\pi}_{jk} = n_{jk}^{-1} \sum_{i=1}^{n_{jk}} y_{ijk}$.

To construct the $(1 - \alpha)\times 100\%$ confidence intervals for the observed data we compute the exact intervals following appropriate guidelines [4]. This is necessary since many space-time locations have just a single observation, i.e., $n_{jk} = 1$, and/or the observed prevalence is at or near 0 (or 1).

Additional References

1. Mielke PW, Berry KJJ. 2007 *Permutation Methods: A Distance Function Approach*. 2nd edn. New York: Springer-Verlag.

2. VanDalen KK, Franklin AB, Mooers NL, Sullivan HJ, Shriner SA. 2010 Shedding Light on Avian Influenza H4N6 Infection in Mallards: Modes of Transmission and Implications for Surveillance. *PLoS ONE* **5**, e12851. (doi:10.1371/journal.pone.0012851)

3. Henaux V, Samuel MD. 2011 Avian influenza shedding patterns in waterfowl: implications for surveillance,. *J. Wildl. Dis.* **47**, 566–78. (doi:10.7589/0090-3558-47.3.566)

4. Hosmer D, Lameshow S. 2008 *Applied Logistic Regression*. New York: Wiley.
