# Supplementary material for: Continental‐scale dynamics of avian influenza in U.S. waterfowl are driven by demography, migration, and temperature
Source: Ecol Appl. 2020 Nov 22;31(2):e2245. doi: 10.1002/eap.2245 (PMC7988533; doi:10.1002/eap.2245)
Supplement: Supplementary file 3 — Appendix S3 [file EAP-31-e2245-s004.pdf]

Supporting Information. Gorsich, E.E., C.T. Webb, A.A. Merton, J.A. Hoeting, R.S. Miller, M.L. Farnsworth, S.R. Swafford, T.J. DeLiberto, K. Pedersen, A.B. Franklin, R.G. McLean, K.R. Wilson, and P.F. Doherty, Jr. 2020. Continental-scale dynamics of avian influenza in U.S. waterfowl are driven by demography, migration, and temperature. Ecological Applications.

APPENDIX S3. ADDITIONAL RESULTS FIGURES AND TABLES

Table S1. Summary of selected logistic regression models from each hypothesis tested. Columns correspond to the hypothesis, number of parameters (np), and the model structure after model selection (Model). All models predict the probability that an individual bird is AIV positive or negative (AIV). The selected model was the Multiple mechanisms model based on AICc (Table 1, main text). Appropriate main effects and lower level interactions were included. Hypothesis numbers refer to their display in Figure 1 and Table 1.

**Hypothesis**	**np**	**Model**
(1) demography	3	AIV ~ age + sex
(2) environmental reservoir	35	AIV ~ flow×temperature polynomial
(3) hot spots	28	AIV ~ latitude×longitude×temperature polynomial
(4) contact network	13	AIV ~ flow + cluster identification + bridging index + MST
(5) multiple mechanisms	64	AIV ~ age + cluster identification + bridging index + MST + flow×temperature polynomial + latitude×longitude×temperature polynomial

Parameter key for table C1.

temperature polynomial		$T_0 + T_0^2 + T_0^3 + \Delta T + \Delta T^2 + \Delta T^3$
where	T_0	weekly average minimum temperature
	ΔT	change in weekly average minimum temperature from previous week
flow		BHY + BAHY + RHY + RAHY
where	BHY	banding flow for hatch-year birds (four-week window)
	BAHY	banding flow for after hatch-year birds
	RHY	recovery flow for hatch-year birds
	RAHY	recovery flow for after hatch-year bird
MST		minimum spanning trees index

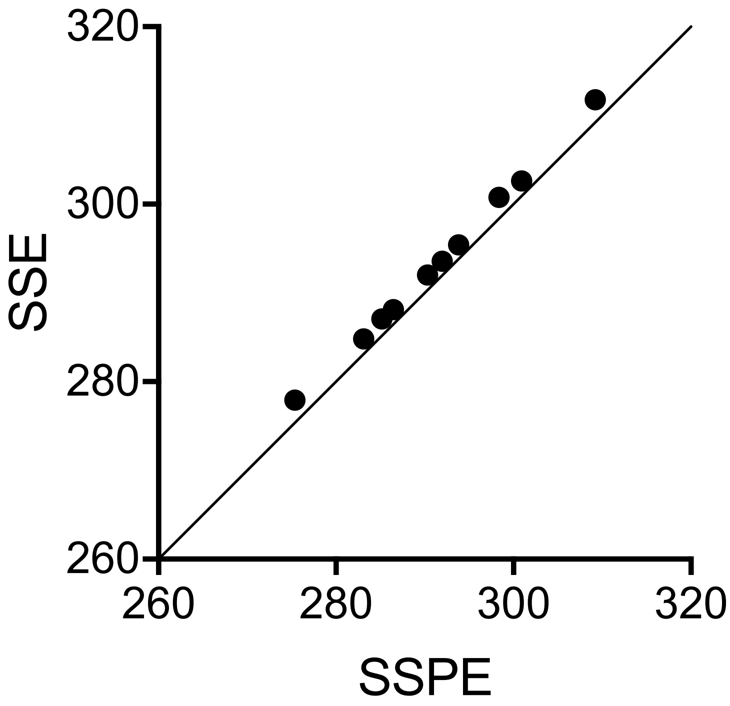

Figure S1. Cross-validation summary plot of error sum of squares (SSE) and pure error sum of squares (SSPE). The 1:1 line is plotted for comparison. To conduct cross-validation, we randomly separated the data into 10 equal partitions. We re-fit the model in Table C3 using nine of the partitions, predicted the response for the excluded partition, and repeated this procedure for all ten combinations to calculate the error sum of squares (SSE) and the pure error sum of squares (SSPE) for each partition. SSPE is calculated by summing the squared difference between each observed response from the averaged observed response at a given predictor value. Because SSPE quantifies the proportion of SSE due to random error rather than lack of fit error, the model is appropriate because the SSPE is a large component of the SSE.

(A)

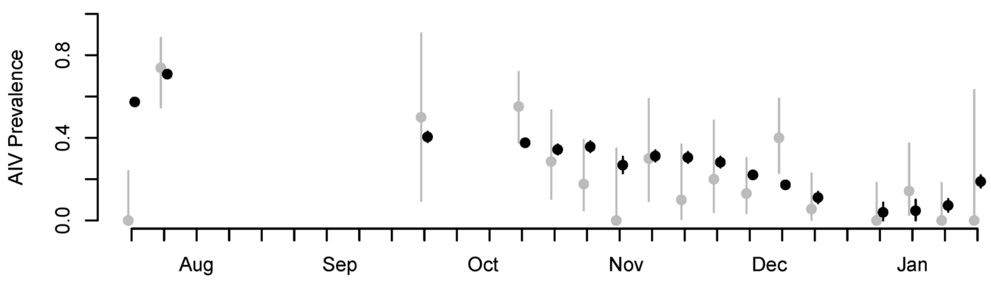

(B)

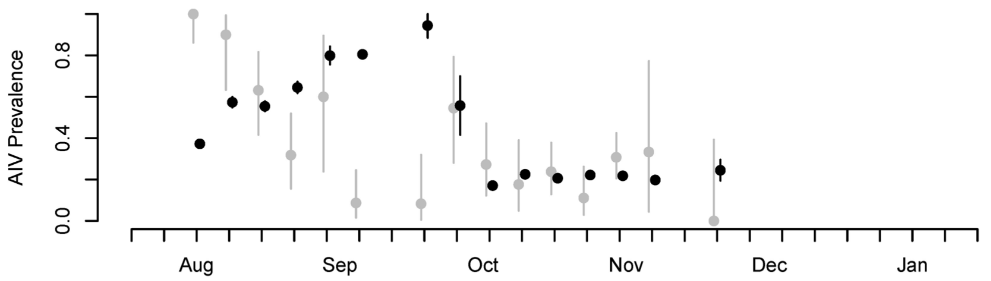

(C)

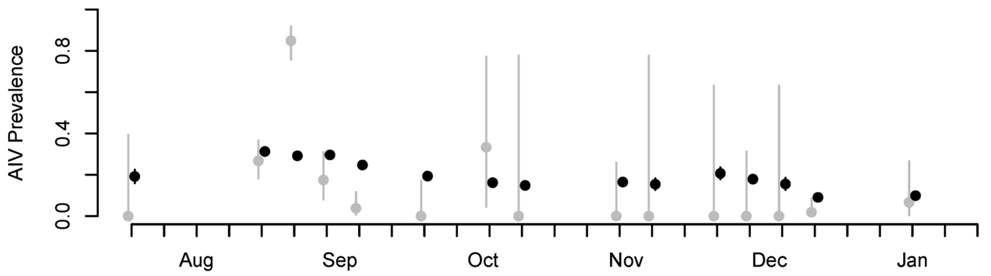

Figure S2. Out-of-sample validation. Comparison between the observed data from 2009 (light grey) and model predictions for 2009 (black) across time at three distinct nodes. Model predictions are based on parameter estimates from fitting to the 2007-2008 data and covariates for the 2009 biological year. (A) Node D in Figure 3 (Pacific Northwest). (B) Node E in Figure 3 (Northern central plains). (C) Node F in Figure 3 (Southern New England).

Table S2. Parameter estimates for the selected model for the multiple mechanisms hypothesis.

	Estimate	Std. Error	z value	Pr(>|z|)	
Intercept	-8.26E+00	1.07E+02	-0.077	0.938	
Age (hatch year)	5.17E-01	4.01E-02	12.909	0.000	***
Northeast Cluster	1.03E+01	1.07E+02	0.096	0.924	
California Cluster	1.05E+01	1.07E+02	0.098	0.922	
West/Pacific Cluster	1.07E+01	1.07E+02	0.100	0.920	
Mississippi Valley Cluster	1.02E+01	1.07E+02	0.096	0.924	
Ohio Valley Cluster	1.02E+01	1.07E+02	0.096	0.924	
Florida Cluster	-8.91E-01	2.52E+02	-0.004	0.997	
Bridging index	3.75E-01	2.07E-01	1.815	0.069	.
T_0	2.99E+00	6.69E-01	4.472	0.000	***
ΔT	4.04E+01	9.44E+00	4.281	0.000	***
T_0^2	-7.33E-02	1.02E-01	-0.718	0.473	
ΔT^2	7.67E+00	1.11E+01	0.694	0.488	
T_0^3	-1.56E-02	6.64E-03	-2.350	0.019	*
ΔT^3	-6.23E+00	3.82E+00	-1.629	0.103	
BHY	5.35E+01	6.44E+01	0.831	0.406	
BAHY	-5.76E+01	5.88E+01	-0.979	0.328	
RHY	2.54E+01	2.39E+01	1.064	0.287	
RAHY	2.57E+01	1.75E+01	1.464	0.143	
Latitude	-4.16E-02	1.25E-01	-0.332	0.740	
Longitude	1.01E-01	5.35E-02	1.891	0.059	.
BHY*T_0	-7.77E+00	2.12E+01	-0.367	0.714	

BAHY*T_0	2.71E+01	2.23E+01	1.216	0.224	
RHY*T_0	7.78E-01	4.96E+00	0.157	0.875	
RAHY*T_0	1.79E+00	7.15E+00	0.250	0.803	
BHY*ΔT	-7.32E+01	2.65E+01	-2.765	0.006	**
BAH*ΔT	3.37E+02	1.08E+02	3.115	0.002	**
RHY*ΔT	5.27E+01	6.17E+01	0.854	0.393	
RAHY*ΔT	-3.57E+01	4.15E+01	-0.861	0.389	
BHY*T_0^2	3.78E+00	2.38E+00	1.593	0.111	
BAHY*T_0^2	-5.80E+00	3.14E+00	-1.848	0.065	.
RHY*T_0^2	-3.03E-01	7.59E-01	-0.399	0.690	
RAHY*T_0^2	-1.22E+00	1.24E+00	-0.981	0.326	
BHY*ΔT^2	-2.36E+02	5.20E+01	-4.533	0.000	***
BAHY*ΔT^2	4.49E+02	1.34E+02	3.340	0.001	***
RHY*ΔT^2	9.11E+01	6.93E+01	1.316	0.188	
RAHY*ΔT^2	-1.75E+02	6.08E+01	-2.881	0.004	**
BHY*T_0^3	-2.31E-01	8.65E-02	-2.673	0.008	**
BAHY*T_0^3	3.37E-01	1.35E-01	2.505	0.012	*
RHY*T_0^3	3.84E-02	8.76E-02	0.439	0.661	
RAHY*T_0^3	-1.80E-01	1.53E-01	-1.174	0.241	
BHY*ΔT^3	-1.01E+02	2.66E+01	-3.795	0.000	***
BAHY*ΔT^3	1.47E+02	4.97E+01	2.952	0.003	**
RHY*ΔT^3	3.62E+01	2.26E+01	1.602	0.109	
RAHY*ΔT^3	-8.04E+01	2.57E+01	-3.125	0.002	**
Latitude*Longitude	-1.73E-03	1.24E-03	-1.396	0.163	

T_0*Latitude	-7.29E-02	1.58E-02	-4.616	0.000	***
ΔT*Latitude	-9.19E-01	2.26E-01	-4.061	0.000	***
T_0^2*Latitude	1.45E-03	2.37E-03	0.612	0.541	
ΔT^2*Latitude	-1.72E-01	2.60E-01	-0.662	0.508	
T_0^3*Latitude	3.91E-04	1.56E-04	2.500	0.012	*
ΔT^3*Latitude	1.41E-01	8.93E-02	1.579	0.114	
T_0*Longitude	2.61E-02	6.57E-03	3.970	0.000	***
ΔT*Longitude	3.94E-01	9.50E-02	4.152	0.000	***
T_0^2*Longitude	-1.19E-03	1.02E-03	-1.169	0.243	
ΔT^2*Longitude	6.41E-02	1.13E-01	0.569	0.569	
T_0^3*Longitude	-1.06E-04	6.41E-05	-1.647	0.100	.
ΔT^3*Longitude	-6.80E-02	3.93E-02	-1.730	0.084	.
T_0*Latitude*Longitude	-6.52E-04	1.55E-04	-4.204	0.000	***
ΔT*Latitude*Longitude	-8.74E-03	2.25E-03	-3.886	0.000	***
T_0^2*Latitude*Longitude	2.52E-05	2.36E-05	1.065	0.287	
ΔT^2*Latitude*Longitude	-1.34E-03	2.64E-03	-0.509	0.611	
T_0^3*Latitude*Longitude	2.71E-06	1.51E-06	1.795	0.073	.
ΔT^3*Latitude*Longitude	1.54E-03	9.11E-04	1.693	0.090	.

Significance codes: 0 '***' 0.001 '**' 0.01 '*' 0.05 '.'
