# Supplementary material for: Continental‐scale dynamics of avian influenza in U.S. waterfowl are driven by demography, migration, and temperature
Source: Ecol Appl. 2020 Nov 22;31(2):e2245. doi: 10.1002/eap.2245 (PMC7988533; doi:10.1002/eap.2245)
Supplement: Supplementary file 6 — Video S1Legend [file EAP-31-e2245-s002.pdf]

Supporting Information. Gorsich, E.E., C.T. Webb, A.A. Merton, J.A. Hoeting, R.S. Miller, M.L. Farnsworth, S.R. Swafford, T.J. DeLiberto, K. Pedersen, A.B. Franklin, R.G. McLean, K.R. Wilson, and P.F. Doherty, Jr. 2020. Continental-scale dynamics of avian influenza in U.S. waterfowl are driven by demography, migration, and temperature. Ecological Applications.

Video S1. Total banding flow at each spatial location animated across a biological year at a monthly time scale. Circle area is proportional to the number of birds banded. The movie illustrates that banding flow captures early season movement.
