# Supplementary material for: Continental‐scale dynamics of avian influenza in U.S. waterfowl are driven by demography, migration, and temperature
Source: Ecol Appl. 2020 Nov 22;31(2):e2245. doi: 10.1002/eap.2245 (PMC7988533; doi:10.1002/eap.2245)
Supplement: Supplementary file 7 — Video S2Legend [file EAP-31-e2245-s001.pdf]

Supporting Information. Gorsich, E.E., C.T. Webb, A.A. Merton, J.A. Hoeting, R.S. Miller, M.L. Farnsworth, S.R. Swafford, T.J. DeLiberto, K. Pedersen, A.B. Franklin, R.G. McLean, K.R. Wilson, and P.F. Doherty, Jr. 2020. Continental-scale dynamics of avian influenza in U.S. waterfowl are driven by demography, migration, and temperature. Ecological Applications.

Video S2. Total recovery flow at each spatial location animated across a biological year at a monthly time scale. Circle area is proportional to the number of birds recovered. The movie illustrates that recovery flow captures later season movement.
